# SARS-CoV-2 and Glutamine: SARS-CoV-2 Triggered Pathogenesis *via* Metabolic Reprograming of Glutamine in Host Cells

**DOI:** 10.3389/fmolb.2020.627842

**Published:** 2021-01-11

**Authors:** Shiv Bharadwaj, Mahendra Singh, Nikhil Kirtipal, Sang Gu Kang

**Affiliations:** ^1^Department of Biotechnology, Institute of Biotechnology, College of Life and Applied Sciences, Yeungnam University, Gyeongsan, South Korea; ^2^Department of Science, Modern Institute of Technology, Rishikesh, India

**Keywords:** glutamine, COVID-19, metabolic reprogramming, hypoxia-inducible factor 1-alpha, glutaminolysis

## Abstract

Severe acute respiratory syndrome coronavirus 2 (SARS-CoV-2) infection, as coronavirus disease 2019 (COVID-19) pandemic, has killed more than a million people worldwide, and researchers are constantly working to develop therapeutics in the treatment and prevention of this new viral infection. To infect and induced pathogenesis as observed in other viral infections, we postulated that SARS-CoV-2 may also require an escalation in the anabolic metabolism, such as glucose and glutamine, to support its energy and biosynthetic requirements during the infection cycle. Recently, the requirement of altered glucose metabolism in SARS-CoV-2 pathogenesis was demonstrated, but the role of dysregulated glutamine metabolism is not yet mentioned for its infection. In this perspective, we have attempted to provide a summary of possible biochemical events on putative metabolic reprograming of glutamine in host cells upon SARS-CoV-2 infection by comparison to other viral infections/cancer metabolism and available clinical data or research on SARS-CoV-2 pathogenesis. This systematic hypothesis concluded the vital role of glutaminase-1 (GLS1), phosphoserine aminotransferase (PSAT1), hypoxia-inducible factor-1 alpha (HIF-1α), mammalian target of rapamycin complex 1 (mTORC1), glutamine-fructose amidotransferase 1/2 (GFAT1/2), and transcription factor Myc as key cellular factors to mediate and promote the glutamine metabolic reprogramming in SARS-CoV-2 infected cells. In absence of concrete data available for SARS-CoV-2 induced metabolic reprogramming of glutamine, this study efforts to connect the gaps with available clinical shreds of evidence in SARS-CoV-2 infection with altered glutamine metabolism and hopefully could be beneficial in the designing of strategic methods for therapeutic development with elucidation using *in vitro* or *in vivo* approaches.

## Introduction

Over the last two decades, several human coronaviruses (HCoVs) have been recorded to cross the species barrier into humans and are known for triggering fatal respiratory diseases, viz. severe acute respiratory syndrome coronavirus (SARS), middle east respiratory syndrome (MERS), and coronavirus disease 2019 (COVID-19) as a pandemic (Kirtipal et al., 2020). Since the outbreak of the first coronavirus, SARS-CoV, extensive research has been conducted to discover HCoVs pathogenesis for the development of therapeutic agents; however, cellular mechanisms that aid HCoVs pathogenesis in the host cells are not yet fully discovered. Under current pandemic COVID-19 — caused by one of the highly infectious HCoVs strain severe acute respiratory syndrome coronavirus 2 (SARS-CoV-2), there is no potential therapeutic agent available against this infection; thereof, it is a potential threat to humans.

**Figure 1:**
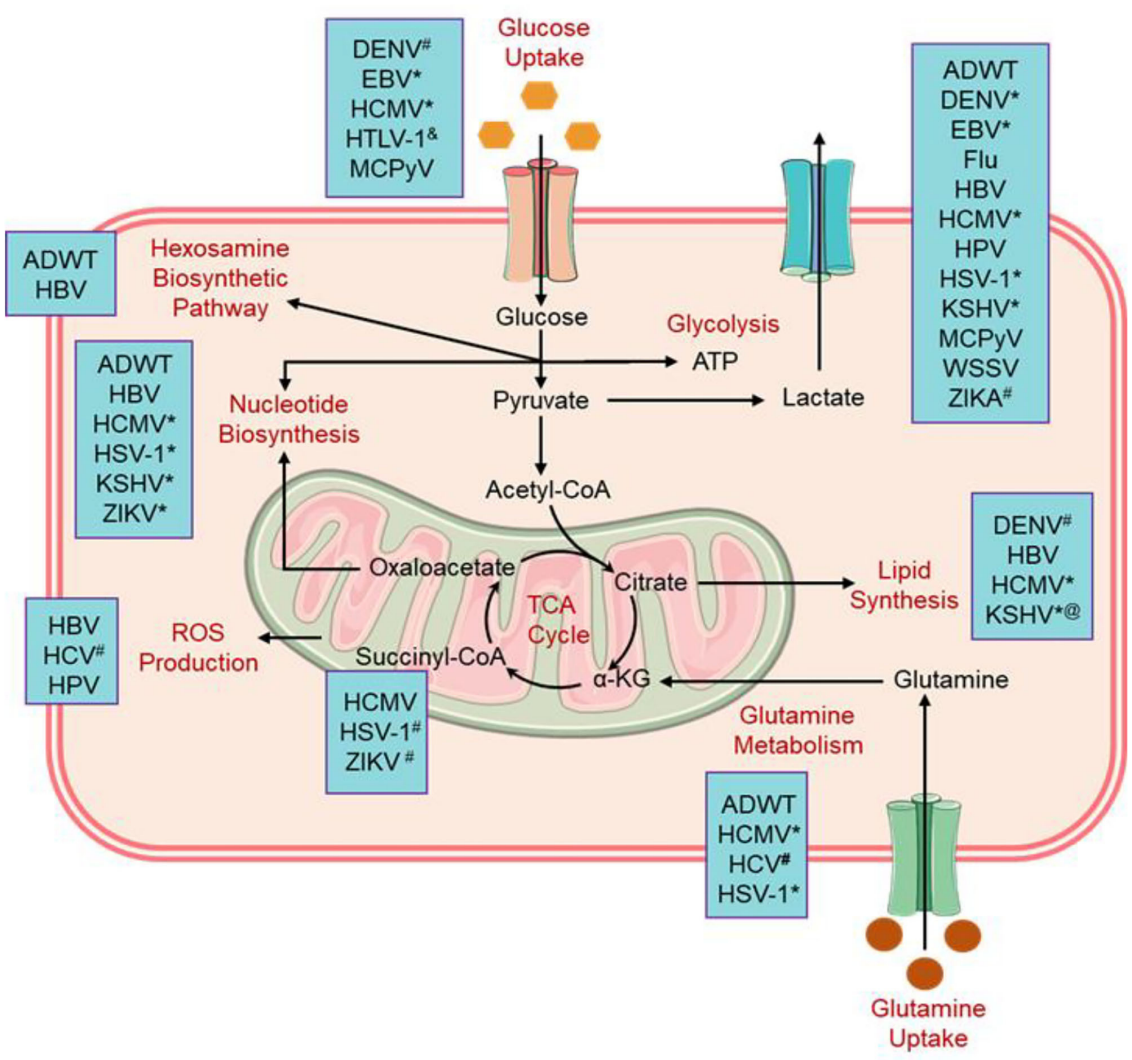
Alternation in the cellular metabolic pathways induced by viral infections. ^#^Flavivirus family; ^&^virus reduced this metabolic activity, ^@^KSHV enhance the lipid synthesis pathway and retarded the synthesis of cholesterol; *Herpesvirus family (Thaker et al., 2019). ADWT, Wild-type adenovirus; DENV, Dengue virus; EBV, Epstein-Barr virus; Flu, Influenza virus; HBV, Hepatitis B virus; HCV, Hepatitis C virus; HSV-1, Herpes simplex virus 1; HPV, Human papillomavirus; HCMV, Human cytomegalovirus; HTLV-1, Human T-lymphotropic virus 1; KSHV, Kaposi's sarcoma-associated herpesvirus; MCPyV, Merkel cell polyomavirus; WSSV, White spot syndrome virus; and ZIKV, Zika virus.

Albeit viruses are well established to alter the host cell metabolism and being studied for over a decade, but the mechanisms and consequences of virus-induced metabolic reprogramming are not yet fully explored in detail. Besides, viruses were elucidated to solely rely on the host cell machinery for aberrant proliferation, i.e., they stimulate anabolism to produce macromolecules required for the replication and assembly of the new virions in the host cell (Figure 1). With the advancement in modern science and constant exploration of virus interactions with the host cells, it is now well documented that virus-induced metabolic reprogramming in the infected cells to continue their ideal biosynthetic demands *via* “pro-viral metabolic changes” (Dyer et al., 2019). Whereas, the infected cells have developed complex metabolic tactics that assist in the inhibition of virus proliferation *via* promoting “antiviral metabolic changes” (Yu and Alwine, 2002; Maynard et al., 2010; Netea et al., 2016). Intriguingly, metabolic traits depicted by a viral infection in the host cell are regularly reflected as the metabolic alternations in cancer cells, including enhanced anabolic metabolism to sustain viral proliferation (Vastag et al., 2011; Thai et al., 2014). In this context, several core metabolic pathways in the host cell, including Warburg effect (aerobic glycolysis), pentose phosphate pathway activation, amino acid catabolism (glutaminolysis — a glutamate-driven anaplerosis), nucleotide biosynthesis, lipid metabolism, and amino acid biosynthesis, were identified as virus-induced alternations from various viral families, reviewed elsewhere (Figure 1) (Sanchez et al., 2015). Hence, requirements in metabolic processes under pathological conditions have been considered as a topic of investigation for therapeutic developments (Smallwood et al., 2017).

**Figure 2:**
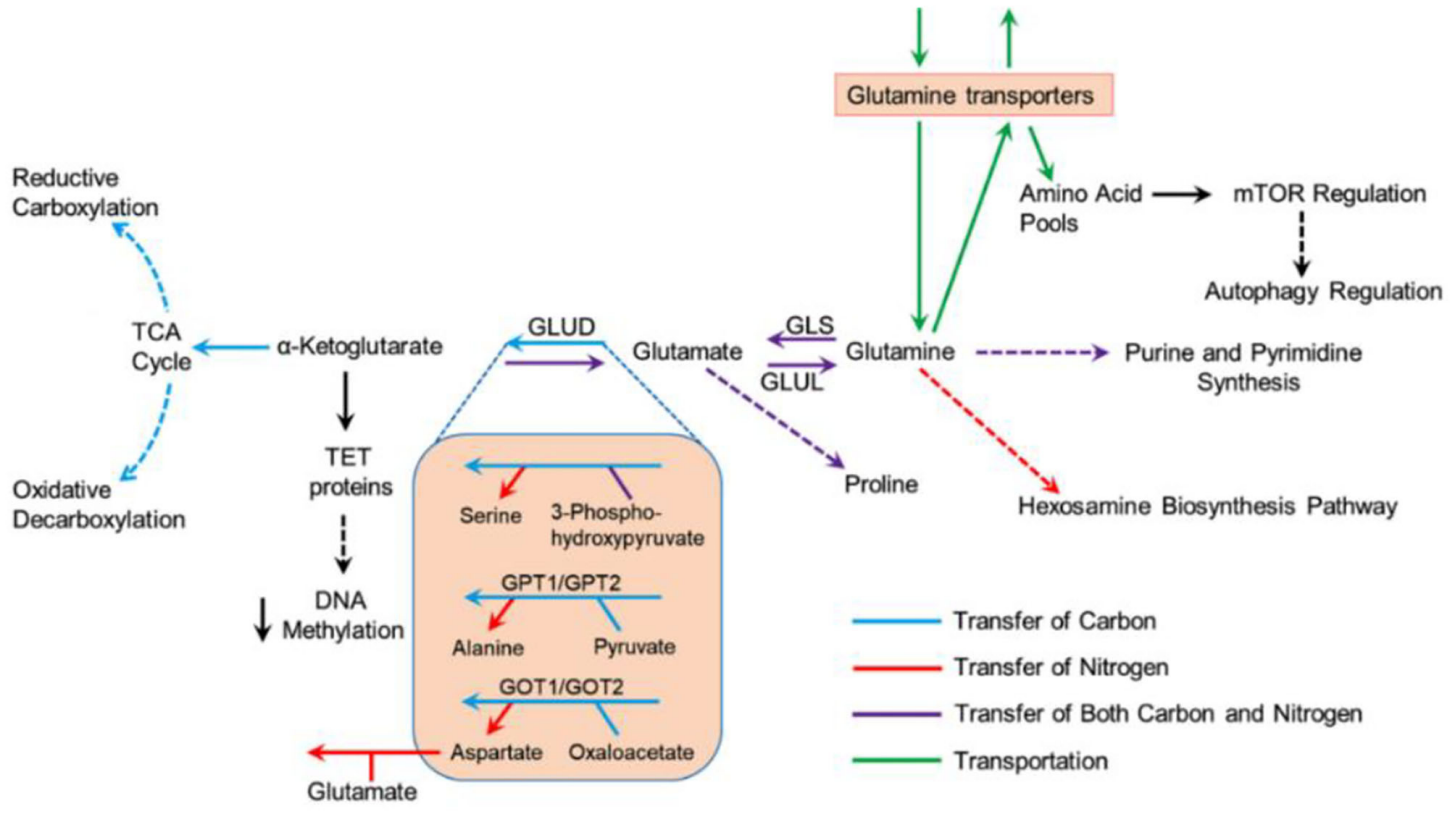
Metabolism of glutamine under multiple biosynthetic pathways in cells (Still and Yuneva, 2017).

Glutamine, the most profuse amino acid in the blood, is needed as a substitute fuel to the tricarboxylic acid (TCA) cycle to produce adenosine triphosphate (ATP). It also contributes to a wide range of biological routes in the cells such as the production of nucleotides, amino acids, and fatty acids. It is noteworthy to mention that glutamine has been noted as an essential factor in the activation of mammalian target of rapamycin complex 1 (mTORC1), reactive hexosamine biosynthesis pathway (HBP), and reactive oxygen species (ROS) homeostasis (Figure 2) (Still and Yuneva, 2017). Moreover, glutamine has been established as a source of fuel to the immune cells, including macrophages and lymphocytes, that are required to produce specific immunostimulatory effects (Ardawi and Newsholme, 1983; Newsholme et al., 1985). Recently, glutamine and glutamate-derived amino acid biosynthesis were demonstrated as an important factor to be required for collagen protein production in fibroblasts and differentiation of myofibroblast in human lung (Hamanaka et al., 2019). As previously reported in several respiratory viruses, SARS-CoV-2 is now known to target the host cells, including myofibroblast, expressing angiotensin-converting enzyme 2 (ACE2) receptors and induction of acute respiratory distress syndrome (ARDS) (Kirtipal et al., 2020). Thereof, based on the available literature, we have discussed the putative role of glutamine under the consequences of induced metabolic reprogramming in SARS-CoV-2 infected cells that essentially contributes to the promotion of virus pathogenesis and reduction in innate immunity of the host body. Currently, researchers are working on therapeutic development against tumor or cancer by targeting glutamine metabolism *via* various potential targets (Kodama et al., 2020; Lee et al., 2020; Yoo et al., 2020); a greater understanding of the distinct roles that glutamine metabolic reprogramming plays in tumors or other viral infected cells as reference for SARS-CoV-2 infection may aid in providing useful insights on SARS-CoV-2 pathogenesis. Thereof, this information can be applied in the advancement of more specific therapeutic strategies to tackle the proliferation of SARS-CoV-2 infection.

## Glutamine and SARS-CoV-2

### Exacerbated Glutamine Metabolism

**Figure 3:**
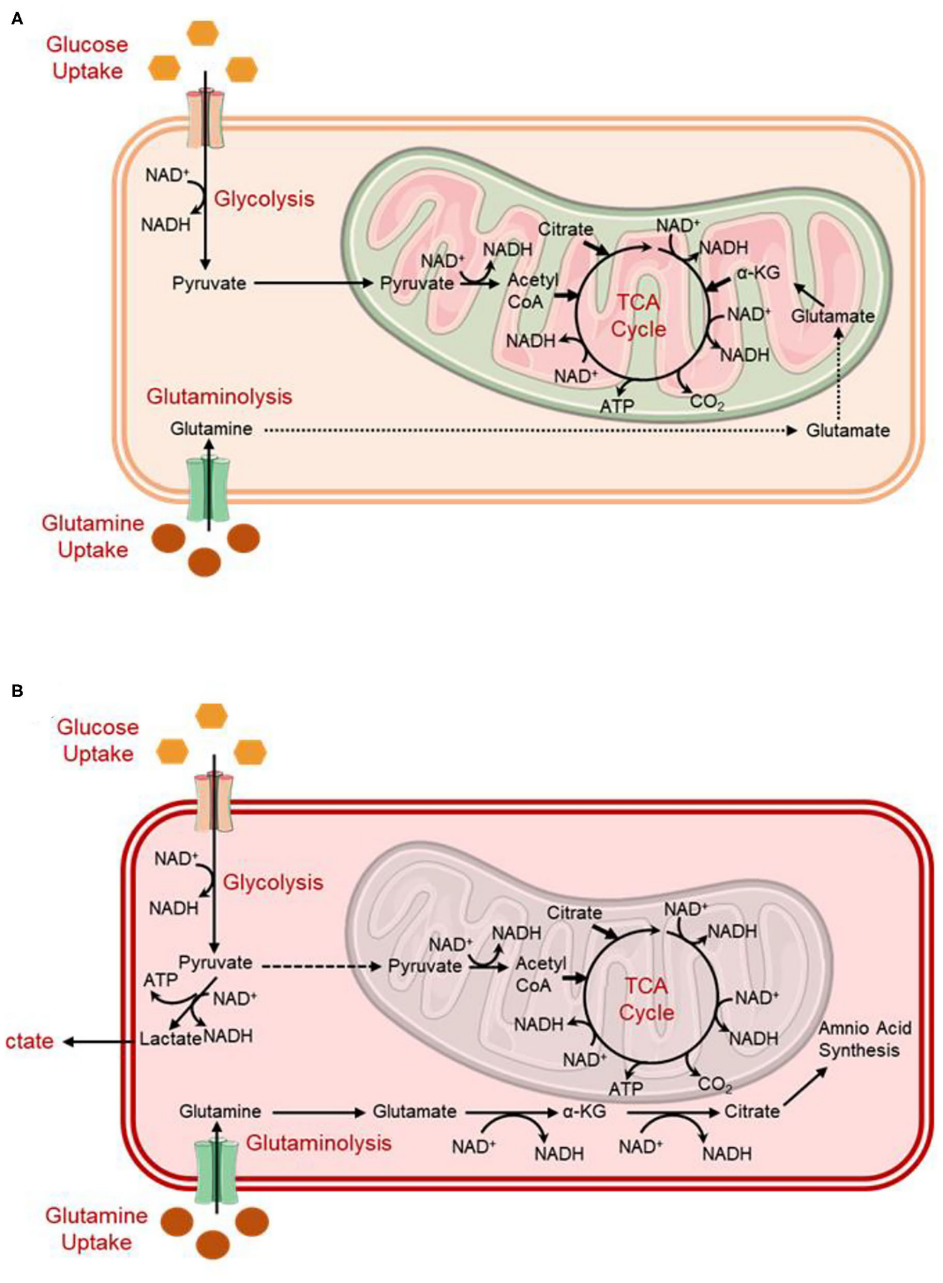
Simplified schematic depicting metabolism in **(A)** quiescent cells compared to **(B)** virally infected/tumor cells (Dyer et al., 2019).

Viruses are specialized intracellular parasites that rely on the falsification and hijacking of host cell metabolism to support both energy and molecular building blocks vital in the generation of viral progenies (Dyer et al., 2019). Recent studies exploring the influence of nutrient deficiency on the virus replication, including Herpes simplex virus 1 (HSV-1), Human cytomegalovirus (HCMV), and poliomyelitis virus, in the virus-infected cells, revealed the importance of both glucose and glutamine as an unlimited source of renewable energy (Eagle and Habel, 1956; Lewis and Scott, 1962). For instance, in absence of exogenous glutamine, vaccinia virus (VACV)-infected cells exhibited a significant decline in viral particle production while depriving infected cells of exogenous glucose had no considerable alternation in virus generation (Fontaine et al., 2014). Likewise, enterovirus 71 (EV71) exhibited similar results in replication corresponds to a change in glutamine/glutamate metabolism (Cheng et al., 2020). However, HCMV demonstrated an excessive dependence on the utilization of both glucose and glutamine for virus replication in infected-cells (Munger et al., 2006, 2008; Chambers et al., 2010; Sanchez et al., 2015). Interestingly, SARS-CoV-2 was also reported to consume excessive glucose for viral replication (Codo et al., 2020), but the effect of glutamine is not yet studied for SARS-CoV-2 infection. Normally, glucose is considered as the main source provider of ATP production in the cell *via* glycolysis and the TCA cycle (Figure 3). However, under chronic conditions like viral infections or cancer where glucose-derived carbon is seized from the TCA cycle, i.e., in aerobic glycolysis or the Warburg effect, glutamine has often been shown to reload the TCA cycle *via* α-ketoglutarate (αKG), a process termed as an anaplerosis (DeBerardinis et al., 2007, 2008; Ahn and Metallo, 2015). Interestingly, SARS-CoV-2 replication and induced responses in infected monocytes were suggested to be prolonged by a switch to aerobic glycolysis or the Warburg effect (Codo et al., 2020). Moreover, glycolysis was monitored to refill the supply of fatty acid synthesis for membrane development during the infection as a substitute for the TCA cycle (Yu et al., 2011). Hence, the elevated demand for fatty acid synthesis in virus-infected cells was suggested to achieve by cataplerosis of citrate derived in the TCA cycle (Figure 3), which is constantly reloaded by glutaminolysis (Yu et al., 2011). In addition, glutaminolysis was also observed as an important source of carbon and nitrogen to complete the biosynthetic requirements of virus proliferation (Thai et al., 2015). Remarkably, these biochemical events were analogous to the variations in metabolic reprogramming as detected upon oncogenesis (Yu et al., 2011); these findings advocated the metabolic needs for virus replication in infected cells as akin to those of propagating cancer cells (Hegedus et al., 2014). In accord with these consequences, upregulated glutaminolysis has been reported in adenovirus-, HCV-, HCMV-, and KSHV-infected cells (Sanchez et al., 2015; Levy et al., 2017). Anaplerotic reactions provide the metabolites to the TCA cycle for the aberrant fabrication of diminishing equivalents that are required in the oxidative phosphorylation (OXPHOS) pathway and anabolic carbons for biosynthesis (Owen et al., 2002; Glick et al., 2014). Recent studies also demonstrated the enhanced catalytic activity of glutaminase (GLS) — a key enzyme required in glutaminolysis, in virus-infected cells (Janke et al., 2011). For instance, human bronchial epithelial cells (NHBE) infected with adenovirus wild-type (ADWT) showed an increase in glutamine utilization and GLS activity (Lewis and Scott, 1962). Later, glutamine tracing studies revealed that glutamine acts as a source of citrate to endure the reductive carboxylation pathway in ADWT infected-cells (Figure 3) (Lewis and Scott, 1962). Meanwhile, CB-839 (Telaglenastat) as a pharmacologic inhibitor of GLS enzyme decreases not only the ideal adenovirus replication but also in multiple viruses, including influenza A virus (IAV) and HSV-1 (Lewis and Scott, 1962). Although, mammalian studies have shown both immune-related benefits and pathogenesis-related effects of the glutamine metabolism (Zhu et al., 2016; Cruzat et al., 2018; Piccaluga et al., 2018), this metabolic pathway primarily implicit to prompt SARS-CoV-2 replication in infected host cells as reported earlier in other viruses (Sanchez et al., 2015; Levy et al., 2017). Thus, the following sections discussed the role of glutamine metabolic reprogramming in the induction of SARS-CoV-2 pathogenesis *via* different routes in the human body.

### Altered Glutamine Metabolism and Aberrant Collagen Production in Virus-Induced Lung Fibrosis

Myofibroblasts are well characterized by significant secretion of extracellular matrix (ECM) and expression of alpha-smooth muscle actin (αSMA) (Phan, 2003), which solely depends on the metabolic reprogramming in the cells (Bernard et al., 2018). Myofibroblasts are known to actively participate in the physiological wound healing process, but their perseverance has been linked with desmoplastic reactions in cancer and pathological fibrosis (Radisky et al., 2007; Duffield et al., 2013). In humans, extensive shreds of evidence are available that support an obvious association between the onset of pulmonary fibrosis and viral infections of the lung (Sheng et al., 2020). Remarkably, based on the biopsy/autopsy analysis in SARS-CoV-2 infected patients, similar physiological consequences were concluded as lung fibrosis (Huang et al., 2020; Menter et al., 2020; Tian et al., 2020).

**Figure 4:**
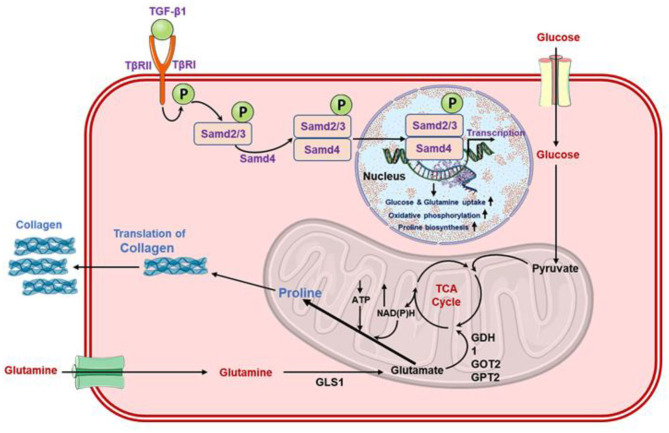
Schematic of the role of glutamine in the activation of TGF-β1 in fibroblasts which upregulated the lung fibrosis via enhanced collagen synthesis (Schworer et al., 2020).

Recently, a specific pathway for glutamine metabolism was established in which glutamine and its conversion to glutamate by GLS enzyme induced the stimulation of transforming growth factor-beta 1 (TGF-β1) which further triggers the differentiation of myofibroblasts from fibroblasts (Figure 4) (Thannickal et al., 2003; Bernard et al., 2018). Of note, glutamate is later processed to αKG by the enzymatic activity of either glutamate dehydrogenase (GLUD), or aminotransferases, such as glutamate-pyruvate transaminases (GPT1 and GPT2), glutamate-oxaloacetate transaminases (GOT1 and GOT2), or phosphoserine transaminase (PSAT1) (Altman et al., 2016; Yang et al., 2017). Also, in addition to anaplerotic reactions, glutamine has been documented to sustain de novo proline production by the activity of Δ^1^ -pyrroline-5-carboxylate synthetase (P5CS), an enzyme decoded by *aldehyde dehydrogenase 18 family, member A1* (*ALDH18A1*) gene. Herein, this enzyme renovates glutamate into pyrroline-5-carboxylate (P5C), which is later processed for the production of proline through P5C reductases (PYCR1, PYCR2, and PYCRL) (Figure 4) (Phang et al., 2015; Hamanaka et al., 2019). Surprisingly, glutamine was not observed in cellular oxygen consumption while oxidative metabolism of αKG by oxoglutarate dehydrogenase (OGDH) was not found as a requirement for the synthesis of collagen protein. Instead, metabolism of glutamate metabolism through PSAT1 and *ALDH18A1*/P5CS was recorded as a requirement in collagen protein synthesis, suggested a major role of glutamine in stimulating the amino acid biosynthesis in matrix production (Hamanaka et al., 2019). Moreover, the altered matrix environment in fibrotic lungs was also noted for bioactive and mechanical characteristics that potentiate profibrotic signaling, and further enhanced the activation of fibroblasts in a feed-forward cycle (Liu et al., 2010; Marinkovic et al., 2013; Parker et al., 2014). Thereof, inhibition of aminotransferases and PSAT1 were suggested as potential targets for therapeutic therapy development against fibrotic diseases (Hamanaka et al., 2019). Furthermore, an enhanced amount of glutamate and a decrease in glutamine concentration was observed in TGF-β1 induced myofibroblasts differentiation against controls, indicated the amplified glutaminolysis in the cells (Bernard et al., 2018). This was coupled with TGF-β1-stimulated GLS isoform (GLS1) expression at both the mRNA and protein levels which lead to the transformation of glutamine into glutamate (Bernard et al., 2018). For instance, glutamine depletion was noticed to diminished TGF-β1-induced manifestation of alpha-smooth muscle actin (α-SMA) and fibronectin as well as inhibited the expression of alpha-1 type I collagen (Col1A1) protein (Bernard et al., 2018). Thus, translation of GLS1 protein by TGF-β1 was suggested to depend on the activation of both small mother against decapentaplegic 3 (SMAD3) and p38 mitogen-activated protein kinase (p38MAPK) pathways (Figure 4) (Bernard et al., 2018), as reported earlier (Derynck and Zhang, 2003; Horowitz et al., 2004; Leask and Abraham, 2004). Moreover, glutaminolysis has been stated to trigger the transcription factor hypoxia-inducible factor-1 alpha (HIF-1α) under a normoxic environment (Kappler et al., 2017). Interestingly, blood monocytes extracted from severe COVID-19 patients also displayed a high-level translation of HIF-1α against healthy donors (Codo et al., 2020). Moreover, HIF-1α is known to controls the manifestation of profibrotic markers downstream to TGF-β1-prompted SMAD3 stimulation (Zhang et al., 2003; Basu et al., 2011). These factors further regulate the multiple genes engaged in metabolic reprogramming (Figure 4) (Semenza et al., 1994, 1996; Hayashi et al., 2004), and standardize the glutamine intermediated redox homeostasis *via* the supervision of GLS1 expression (Stegen et al., 2016). However, under a physiological oxygen tension environment, HIF-1α stabilization is attributed to the inhibition of prolyl hydroxylases (PHD) by hypoxia or mitochondrial ROS which initiated the proteasomal degradation of HIF-1α (Page et al., 2008; Bernard et al., 2015; Burr et al., 2016). Of note, succinate and fumarate as metabolites derived from the TCA cycle have been described in cancer cells to block PHD activity which contributes to the stabilization of HIF-1α (Briere et al., 2005; Selak et al., 2005; Koivunen et al., 2007; Arts et al., 2016). Equivalent results were also reported for SARS-CoV-2 infected monocytes which exhibited succinate oxidation for the stabilization of HIF-1α (Codo et al., 2020). It is important to mention that lung fibroblasts treated with TGF-β1 demonstrated steady-state levels of succinate and fumarate, which were observed to provide stability to HIF-1α at 48 h post-TGF-β1 treatment under normoxia or relative hyperoxia; these conditions were correlated with the expression of GLS1 in the cells (Bernard et al., 2018). Therefore, these results indicated that TGF-β1-stimulated glutaminolysis *via* augmentation of the TCA cycle derived metabolites levels, i.e., succinate and fumarate, imitates the sustained activation of HIF-1α at post 6 h of TGF-β1 treatment (Bernard et al., 2018). Besides, a marked inhibition of HIF-1α induction was recorded with the knocking of GLS1 expression; this supports the downstream consequences of persistent HIF-1α induction in TGFβ1/GLS1-dependent myofibroblasts activation and differentiation (Bernard et al., 2018). Therefore, TGF-β1-stimulated glutaminolysis was suggested to supports myofibroblast functions through gratifying respective bioenergetic requirements by an upsurge in OXPHOS (cataplerosis) and biosynthetic demands *via* supplying anabolic carbons (anaplerosis) (Bernard et al., 2018). It was hitherto reported that differentiation of myofibroblast requires metabolic reprogramming typified by an upsurge in OXPHOS and glycolysis (Figure 4) (Bernard et al., 2015). Hence, glutaminolysis was suggested to be deemed to an amphibolic process, acting as both anabolic and catabolic functions, during differentiation of myofibroblast (Bernard et al., 2018).

Recently, GLS activity was documented as an essential factor to support the ideal replication of IAV in primary normal and diseased bronchial epithelial (NHBE and DHBE) cells, indicated that GLS inhibition by pharmacological inhibitors decreases the replication of multiple viruses (Thai et al., 2015). Besides, glutamine utilization and production of glutamate by GLS enzyme activity were also documented as standard conditions for the optimal replication of adenovirus in primary NHBE cells (Thai et al., 2015). Based on available facts, it was advocated that targeting the glutaminolysis pathway may be considered as an efficient therapeutic approach in ameliorating the progression of fibrotic diseases as recorded in SARS-CoV-2 infection (Huang et al., 2020; Menter et al., 2020; Tian et al., 2020).

### Altered Glutamine Metabolism Donate Substrate in Virus Triggered Hyaluronan Synthesis

**Figure 5:**
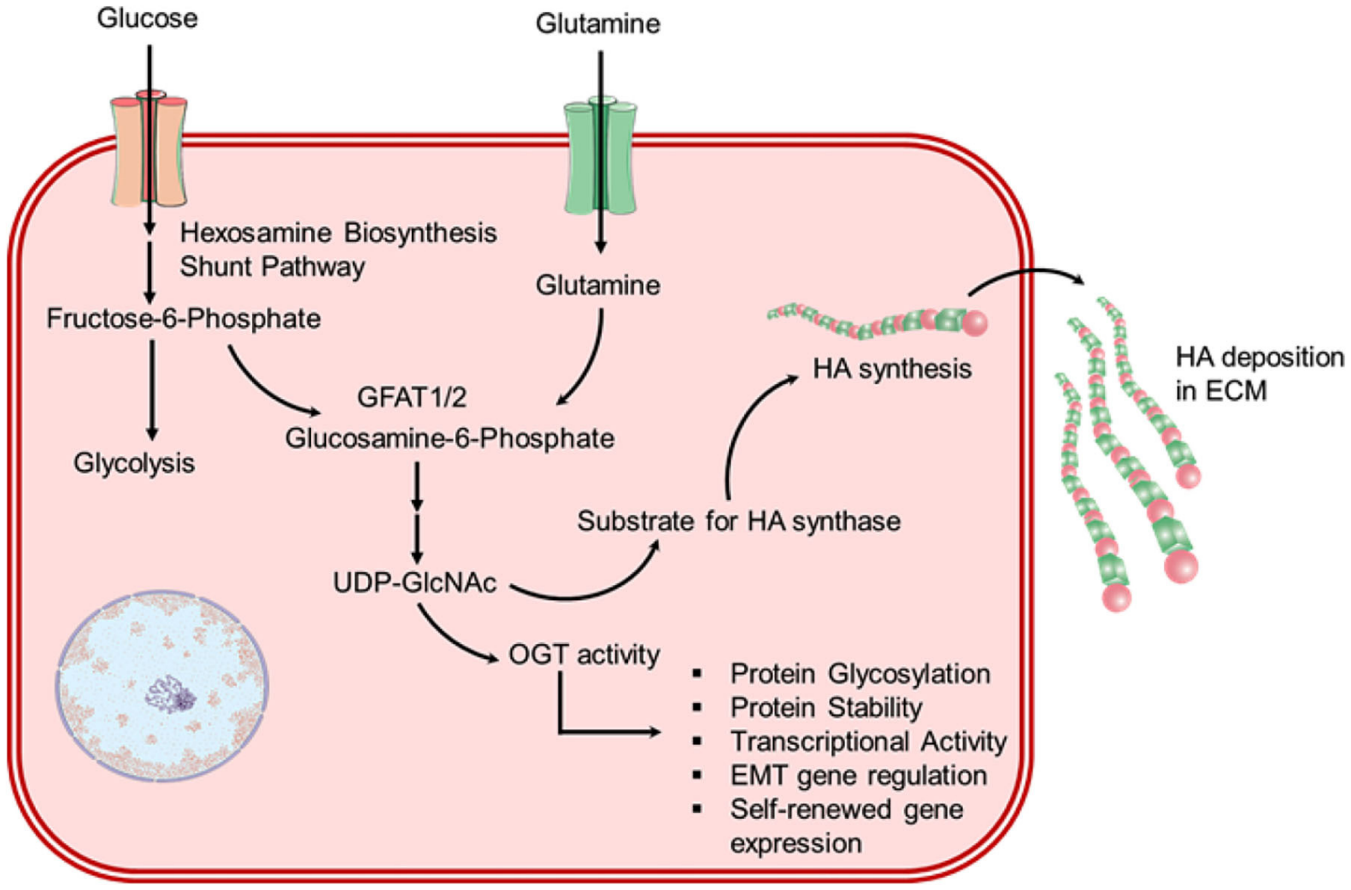
Schematic for overactivated hexosamine biosynthesis pathway (HBP) in chronic virus-infected cells which results in enhanced HA biosynthesis.

Glutamine is utilized to produce the amino acids and hexosamine pathway (HBP) intermediates (Lewis and Scott, 1962). In HBP, glutamine provides a nitrogen atom by an amino group in the transition of fructose-6-phosphate to glucosamine-6-phosphate, and by delivering carbon atoms for the transition of glucosamine-6-phosphate to N-acetylglucosamine (GlcNAc)-6-phosphate *via* generation of acetyl-CoA from citrate (Figure 5). Herein, both glucose and glutamine are requisite for O-linked and N-linked glycosylation, which is important for both protein function and stability (Still and Yuneva, 2017). The consumption of both glucose and glutamine by HBP has been reported to control the signal transduction by the glycosylation of the interleukin-3 receptor that further synchronizes the cell development and propagation (Wellen et al., 2010). In the recent study, NHBE cells infected with ADWT showed enhanced intracellular pool sizes of HBP intermediates and transcript levels of HBP enzymes, including hexokinase 2 (HK2) (Thai et al., 2014). Later, a Myc-dependent change in hexosamine biosynthesis in infected cells with adenovirus was also observed (Thai et al., 2015). Moreover, Myc activity has been well studied with regulating the absorption and metabolism of glutamine in cancers *via* three distinct pathways; (i) Myc directly influence the expression of amino acid transporter proteins, such as SLC5A1 and SLC7A1 (Gao et al., 2009), (ii) Myc induced the production of glutamate from glutamine *via* indirectly mediating GLS expression, i.e., Myc transcriptionally inhibits the microRNAs (such as miR-23a and miR-23b) that attach to the 3′ untranslated region (UTR) of *GLS* mRNA and encourage its degradation (Gao et al., 2009), (iii) Myc enhanced the translation of several enzymes required in nucleotide biosynthesis from glutamine (Dang, 2010; Wise and Thompson, 2010). For instance, cancer cells exhibited 10-folds uptake of glutamine against any other amino acid, and restriction of glutamine to transformed cells was noticed with apoptosis (Yuneva et al., 2007; Wise et al., 2008). Interestingly, HBP delivers the uridine diphosphate N-acetylglucosamine (UDP-GlcNAc) (Figure 5), an essential substrate for the synthesis of hyaluronan or hyaluronic acid (HA) — a polymer of glucuronic acid (GlcUA) and N-acetylglucosamine (GlcNAc), orchestrated by HA synthases (HAS1-3) (Vigetti et al., 2012; Bohaumilitzky et al., 2017; McCarthy et al., 2018). HA, a pervasive component in the microenvironment of cells, has been reported for critical effects on cell behavior under both physiological and pathological conditions (Jiang et al., 2011). For instance, HA synthesis in tumors is strictly linked with the metabolic level of the cells and regulated by the glutamine-fructose amidotransferase 1/2 (GFAT1/2) (Sharma et al., 2020). Remarkably, enhanced HBP was observed in several cancers, and its inhibition by either preventing consumption of glutamine [by glutamine analog, 6-diazo-5-oxo-L-norleucine (DON)] or targeting GFAT1/2 results in deterioration of tumor (Li et al., 2017; Asthana et al., 2018; Zhang et al., 2018). Besides, a number of studies have specified the pivotal function of HA in human respiratory disease, where viral infections result in the noticeable and rapid production of HA (Lauer et al., 2015; Johnson P. et al., 2018; Bell et al., 2019). On this note, an autopsy of SARS-CoV-2 infected patients also demonstrated a fluidic substance filled in the lungs that are not yet defined but HA was anticipated to cause hypoxia conditions in the lungs and breathless symptoms in SARS-CoV-2 infected patients (Hellman et al., 2020; Shi et al., 2020; Xu et al., 2020). Remarkably, HA accumulation was documented in patients with ARDS (Hallgren et al., 1989; Modig and Hallgren, 1989), which is also predicted in SARS-CoV-2 infection (D'Abramo et al., 2020; Kirtipal et al., 2020). Moreover, SARS-CoV-2 infected cells were suggested to be defective for the production and regulation of HA (Shi et al., 2020); additionally, high level of tumor necrosis factor (TNF) and interleukin-1 (IL-1) in the lungs of SARS-CoV-2 infected patients were considered as strong inducers of epithelial cell adhesion molecule (EpCAM^+^) in lung alveolar epithelial cells, HA-synthase 2 (HAS2) in the cluster of differentiation 31 (CD31^+^) endothelium, and fibroblasts (Bell et al., 2019; Shi et al., 2020). A recent study documented that SARS-CoV-2 infection in bronchoalveolar cells results in enhanced production of HA and glycosaminoglycan (Andonegui-Elguera et al., 2020). Besides, in addition to selectins and integrins, circulating monocytes were demonstrated to adhere to HA by interaction with a cluster of differentiation 44 (CD44) (Hascall et al., 2004). Therefore, the collection of HA on the monocytes was suggested to act as a proinflammatory signal which may assist to elucidate the participation of HA in inflammatory processes (Vigetti et al., 2012). Hence, owing to the central role of HBP in HA synthesis — a key component of the cancer extracellular matrix (ECM), which is considered to be solely dependent on glutamine metabolism (Son et al., 2013), can be targeted to tackle the viral infection. Moreover, as ECM contributes to the maintenance of immune landscape within a tumor and controls the circulation of T cells against tumor through the generation of a 3D matrix and a chemokine gradient (Hartmann et al., 2014), robust ECM has been documented to prevent the specific cells to execute their cytotoxic activity against tumor (Sharma et al., 2020). Based on this reported literature, we hypothesized that similar metabolic changes in SARS-CoV-2-infected cells may lead to the establishment of a microenvironment rich in HA, as suggested earlier (Shi et al., 2020). Such extracellular matrices can restrict the infiltration of cytotoxic T cells as reported in tumor cells (Sharma et al., 2020), and hence, postulated to assist in the proliferation of SARS-CoV-2 infection. Thus, remodeling of the ECM by disturbing HBP can promote activation of macrophages along with infiltration and migration of T cells into the viral infected cells microenvironment which can be used as a potential therapeutic approach against SARS-CoV-2 infection.

### Glutamine Role in Regulating Immune Responses Under Virus Infection

**Figure 6:**
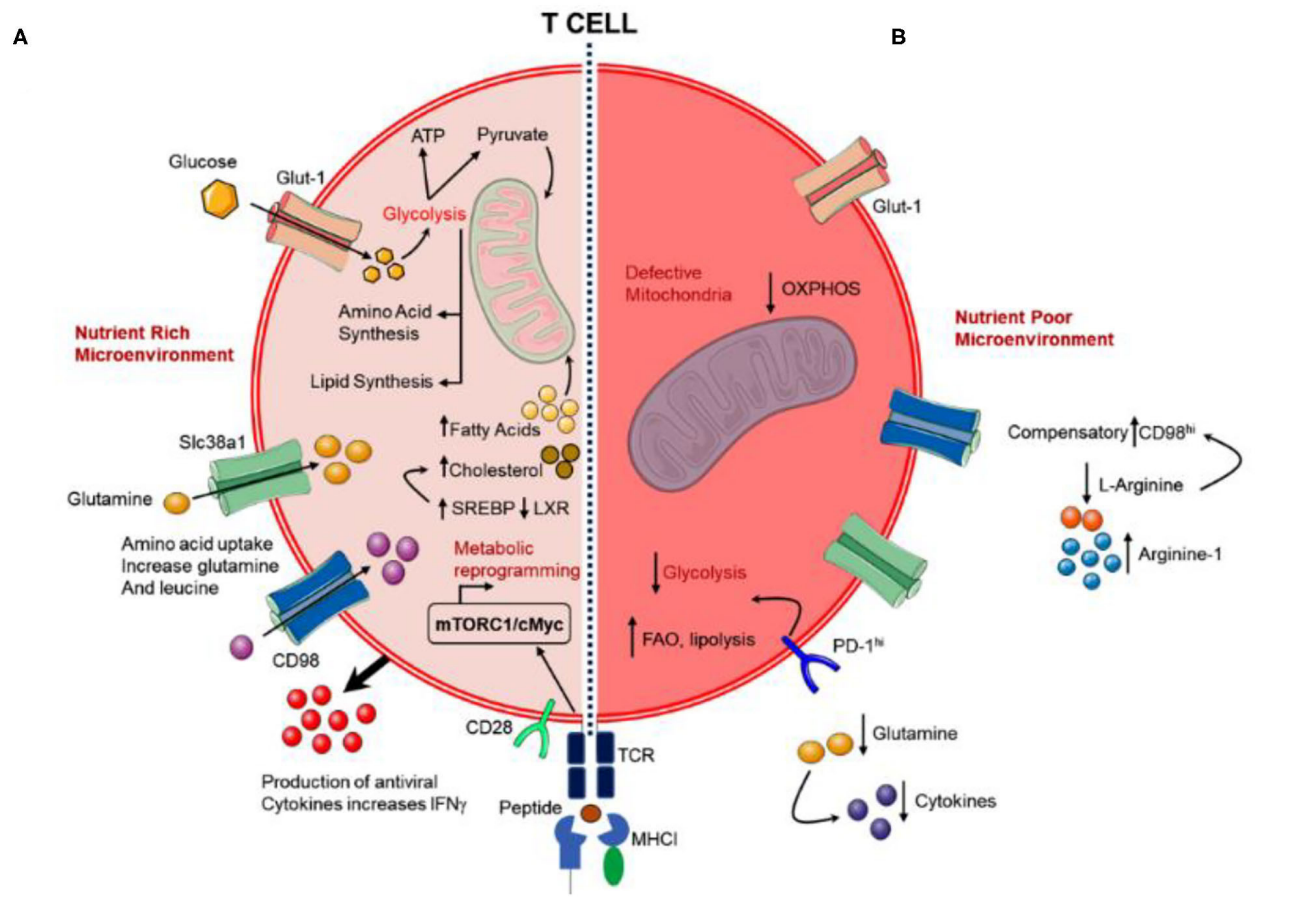
Schematic for **(A)** functional T cell response to viral infection under the nutrient-rich microenvironment and **(B)** exhausted T cell response under chronic viral infections with the nutrient-poor microenvironment.

Viruses can alter the metabolism of both infected-cells and neighboring cells (Yogev et al., 2017), results in functional and phenotypic variations that influence the anti-viral response of T cells (Figure 6) (Kedia-Mehta and Finlay, 2019). There are several evidences that contest for nutrients in immune cells is pertinent at sites of pathogen infections (Kedia-Mehta and Finlay, 2019). Moreover, metabolic rates were also reported with an increment in T cell number following the activation of immune system for proliferative expansion and initiation of effector activities, including aggressive production of cytokines — a process that required considerable levels of energy and cellular biosynthesis (Kedia-Mehta and Finlay, 2019). This results in an enhanced requirement for nutrients such as glucose and glutamine to fuel both biosynthetic and bioenergetic pathways (Figure 6) (Sinclair et al., 2013; Buck et al., 2015). Albeit glucose is a vital substrate, essential amino acids are used in the biosynthesis of protein and nucleotide. For example, the availability of glutamine is critical for the functional activity of mTORC1; stimulated T cells enhanced the activity of members from the glutamine transporter family, including SLC38A1 and SLC1A5, steady with energetic consumption of exogenous glutamine (Bar-Peled and Sabatini, 2014; Nakaya et al., 2014). The activation of mTORC1 further assist in the activation of Myc and HIF-1α (Waickman and Powell, 2012) and mediates the expression of progression genes, *viz*. cyclin A, CDK2/4, and cdc25a, required in T cells (Wang et al., 2011) along with a translation of activation markers and cytokines, including TNF-a, IFN-g, OX40, TIM3, CD137, and Granzyme B (Phan et al., 2016; Palazon et al., 2017). Besides, Myc was also found to accelerate the glutaminolysis in CD8^+^ T cell after activation (Wang et al., 2011; Waickman and Powell, 2012) and enhanced the expression of glutamine transporters, such as SLC32A1 and SLC32A2, needed for the transportation of glutamine into activated T cells (Gnanaprakasam and Wang, 2017). Moreover, deprivation of glutamine from the microenvironment has been reported with impairments in both T cell proliferation and effector function (Carr et al., 2010; Wang et al., 2011; Sinclair et al., 2013). Besides, deficiency or absence of glutamine precluded the production of cytokines and propagation of both Th1 and Th17 cells, and as an alternative endorsed Treg generation (Johnson M. O. et al., 2018). More specifically, SLC1A5 defect on stimulated CD4^+^ T cells was recently demonstrated with reduced uptake of glutamine upon T cell receptor (TCR) engagement and linked with a compromised ability to operate OXPHOS aptly, subsequently results in defective cellular differentiation *in vitro* and *in vivo* (Nakaya et al., 2014). Although previous studies have estimated the T cells, macrophages, and monocytes, etc. as a source for the exudation of cytokines, including IL-6, IL-10, and TNF-α (Minciullo et al., 2016; Kany et al., 2019); analysis of cytokines in SARS-CoV-2 infection suggested that these cytokines were not originated from T cells (Diao et al., 2020). Hence, the source of cytokines production under SARS-CoV-2 infection is one of the topics that demand further investigation. It is progressively recognized that the metabolism of T cell may be compromised during chronic viral infections and in tumors, thereby produced a state of T cell “exhaustion” which precludes T cells to exert their effector function (Figure 6) (Doedens et al., 2013; Chang et al., 2015; Ho et al., 2015). This may be acutely difficult when T cells experienced hypoxic microenvironments (Doedens et al., 2013). Interestingly, similar functional exhaustion was reported in T cells infected by SARS-CoV-2 through monitoring of PD-1 levels under high glucose level, an essential indicator of T cell exhaustion (Codo et al., 2020; Diao et al., 2020). Of note, T cells culture depleted to glutamine exhibited less susceptibility to HIV infection (Clerc et al., 2019; Geltink, 2019); thus, a similar effect needs to monitor for SARS-CoV-2 infection. It is now detailed that metabolic alternations are triggered by recognition of virus particles through Toll-like receptors (TLRs) which subsequently enhanced the transcription factor Myc of the host cell as a cellular response against infection (Bajwa et al., 2016; Smallwood et al., 2017). For instance, viruses that changed Myc expression levels such as polyomavirus (Zullo et al., 1987; Klucky et al., 2004), IAV (Smallwood et al., 2017) parvoviruses (Li et al., 2005), cytomegalovirus (Boldogh et al., 1990), and human immunodeficiency virus (Wen et al., 2005); viruses translating proteins that modify Myc protein stability or activity (Yeh et al., 2004; Awasthi et al., 2005; Kalra and Kumar, 2006); or viral encoded proteins that change Myc mRNA transportation into the cellular cytoplasm and its stabilization (Higashino et al., 2005). Intriguingly, metabolic changes mediated by Myc have often been observed with a state of glucose and glutamine addiction in cancer cells, where malignant cells showed a total reliance on these nutrients to survive (Wise et al., 2008; Smallwood et al., 2017). In this context, the restriction of both glucose and glutamine in IAV infected NHBE exhibited a significant reduction in cell viability (Smallwood et al., 2017). Likewise, it was demonstrated that the Myc-dependent upregulation of GLS activity and glutamine utilization was essential for optimal replication of adenovirus in primary lung epithelial cells, and GLS function was required for ideal HSV-1 and IAV replication (Thai et al., 2015). Conversely, temporary or episodic inhibition of GLS was suggested to program T cells to performed enhance IFNγ-specific effector activities or T cells epigenetic reprogramming to improve the effector function and immunotherapy (Johnson M. O. et al., 2018). These findings hold coherence with SARS-CoV-2 infection which results in T cell dysfunction and lymphopenia (Codo et al., 2020; Zhao et al., 2020). Collectively, it was suggested that the modulation of T cells by regulation of glutamine metabolism in virus-infected cells can be used to provide an alternative approach to therapeutically target T cell function against viral infections.

## Outlook and Conclusion

Although diverse metabolic changes have been reported in several viral infections, but many of the factors involved in the molecular mechanism to induced pathogenesis in the host cell remain unknown. For example, in EBV infection, several studies have documented the importance of viral proteins' relation with 5′ adenosine monophosphate-activated protein kinase (AMPK) and Myc (Lo et al., 2017). Likewise, SARS-CoV-2 main protease was showed to inhibits the expression of IFN-1 in the human kidney epithelial cells (Lei et al., 2020). Recent studies also suggested the pleiotropic effect of nonstructural protein 1 (nsp1) in SARS-CoV-2 to block the host translational machinery and prevent the expression of immune genes (Lei et al., 2020; Thoms et al., 2020; Xia et al., 2020). Thus, hijacking of host cell machinery by SARS-CoV-2 needs to be established through identifying the viral gene products which may interact with essential factors in the host cell to modulate glutamine metabolic reprogramming and may deepen our understanding of viral-induced alternations in host metabolism for viral tropism. Of note, this study presents a comparative analysis of glutamine metabolic changes reported in other viral infections and cancer cells against SARS-CoV-2 infection, suggested the modulation of GLS1, PSAT1, HIF-1α, mTORC1, GFAT1/2, and Myc factors as a conceivable approach to modulate the glutamine metabolism for the inhibition of SARS-CoV-2 pathogenesis. However, the role of these cellular factors is currently not validated by experiments and hence, might be helpful to decipher the glutamine metabolic reprogramming in SARS-CoV-2 infected cells. Although, one report found the overexpression of glycolysis enzymes and HIF-1α in monocytes infected with SARS-CoV-2 (Codo et al., 2020), but this study likely implying the essential role of enzymes and transcription factors involved in glutamine metabolism. Moreover, epigenetic modulation of transcription factors such as mTORC1 and Myc-driven metabolism of glutamine in virus activated or infected cells can also be considered. Hence, a comparative proteomic analysis of SARS-CoV-2 infected cells vs. normal cells may provide further insights into the potential differences produced under glutamine metabolic reprogramming.

Though several glutamine metabolism-related therapeutic agents are currently being established against several types of cancer, a similar approach can also be applied against chronic viral infections such as SARS-CoV-2. For instance, therapeutics inhibition of glutamine transporters or GLS enzyme can also be used as an effective method against glutamine-dependent viral infections, irrespective of the precise need for glutamine, as they will block all the metabolic pathways that require glutamine. Hence, inhibition of the target at the precise step that the virus needs should be employed to decrease the wider toxicity and off-target effects, especially in healthy cells that also need glutamine for other essential pathways.

Metabolic alterations in virus-infected cells and tissues are sophisticated networks where efflux of glutamine is essentially expected to sustain a wide range of different pathways, effective energy production, cell signaling, and ROS homeostasis, as in other virus-infected cells or cancer. Likewise, the requirement of glutamine may greatly vary from one cell to another: for example, amino acid requirements in fibroblasts and T-cells upon viral infections. Thus, a full scenario of glutamine metabolism requirement for initiation or progression of SARS-CoV-2 in different tissues is required to be decipher for the development of potential antiviral therapies by targeting glutamine metabolism with limited scope for off-target effects and viral resistance against them.

## Data Availability Statement

The original contributions presented in the study are included in the article/supplementary material, further inquiries can be directed to the corresponding author/s.

## Author Contributions

SB: conceptualization, formal analysis, writing—original draft preparation, and writing—review and editing. SB and NK: methodology and data curation. SB, NK, and SK: validation and investigation. MS and SB: resources. SB and MS: visualization. SK: supervision. All authors have read and agreed to the published version of the manuscript.

## Conflict of Interest

The authors declare that the research was conducted in the absence of any commercial or financial relationships that could be construed as a potential conflict of interest.
